# Inequality in Peruvian neonatal mortality generated by poverty and education, 2011-2019

**DOI:** 10.17843/rpmesp.2022.392.10629

**Published:** 2022-06-30

**Authors:** Jeannette Giselle Ávila Vargas-Machuca

**Affiliations:** 1 National Center for Epidemiology, Prevention and Control of Diseases of the Ministry of Health. Lima, Peru. National Center for Epidemiology Prevention and Control of Diseases of the Ministry of Health Lima Peru

**Keywords:** Health Inequities, Social Determinants of Health, Educational Status, Poverty, Peru

## Abstract

**Objective:**

This study aimed to analyze inequality in the neonatal mortality rate (NMR) between departments in Peru, generated by poverty and education, in the years 2011 and 2019.

**Materials and methods:**

Ecological study based on the analysis of social inequalities in health, recommended by the World Health Organization. The health indicator was the NMR. Poverty, measured as the existence of at least one unmet basic need per department, and education, average years of study of women of childbearing age per department, were selected to stratify equity. We calculated the absolute inequality gap (AG), the relative inequality gap (RG) and the health concentration index (HCI).

**Results:**

A higher NMR was found in departments with greater poverty and less education. In the NMR generated by poverty, the AG decreased from 8.13 to 2.24 between 2011-2019 and the RG from 2.08 to 1.31. The AG of the NMR according to education dropped from 4.50 to 2.31 and the RG from 1.62 to 1.28. The HCI registered values close to zero and with a decreasing trend; in 2019 it was 0.07 for poverty and 0.06 for education.

**Conclusions:**

There is inequality in neonatal mortality between departments in Peru according to poverty and education, which decreased between 2011 and 2019 mainly in the poor or less educated population. The Ministry of Health should continue to reduce neonatal mortality by promoting interventions with a greater population focus.

## INTRODUCTION

Peru is a country of major inequalities and with well-defined groups, either by geographical location or by their accessibility to economic and political resources. The most disadvantaged populations are characterized by being the poorest, are from rural areas, less educated, indigenous and with other unfavorable social conditions with less possibilities of having health care when they need it, access to quality education, work or decent housing ^1^. Social inequalities generate gaps in the chances of survival of newborns, slowing down the sustainable progress that seeks to ensure that children can exercise their right to survive and grow ^2^ ^,^ ^3^.

Peru made significant progress in reducing infant mortality until 2013, which allowed to meet the Millennium Development Goals, thanks to improved coverage of specific interventions such as family planning, prenatal care and qualified birth assistance, which increased in poor and rural areas; in addition to Peru’s economic growth and poverty reduction between 1990 and 2010 ^4^ ^-^ ^6^.

Currently, infant mortality is declining slowly and neonatal mortality is its most important component. Neonatal death is a frequently event, more than 5500 deaths annually at the national level, where 30% of deaths occur in newborns of good weight, at term and without lethal congenital malformations (preventable neonatal mortality). In addition, the national neonatal mortality rate (NMR) is stagnant at 10 per 1000 live births since 2014 and is higher in departments with greater poverty, less education, greater rurality and departments located in the jungle and central highlands regions ^7^; therefore, this event needs to be addressed with a focus on equity in public policies and national programs.

Goal 3 of the Sustainable Development Goals “Ensure healthy lives and promote well-being for all at all ages” is aimed at ensuring health and well-being by improving reproductive, maternal and child health, among others, leaving no one behind and seeking to build a true partnership for development where all countries participate, therefore, inequalities in health indicators should be measured, monitoring trends over time and establishing strategies to promote equity ^8^ ^,^ ^9^. Continuous monitoring of inequalities in NMR and newborn intervention coverage should be a key aspect of any strategy to reach all mothers and their newborns ^10^ ^,^ ^11^.

Previous national studies explored inequality in neonatal mortality generated by poverty, but did not evaluate education. In the period 1999-2001, Huicho *et al* found that the NMR for the least poor and poorest quintiles was 7.8 and 21.9 deaths per 1000 live births, respectively, while for the period 2011-2013 the NMR was 9.1 and 11, showing that the NMR decreased in poorer and rural groups because of the economic growth and the decrease of poverty in Peru ^6^. Tam *et al*, estimated the NMR as of 2017 by eliminating inequalities in the distribution of coverage of maternal, newborn and child health interventions among wealth quintiles, finding that, if the coverage of the poorest quintile were equal to the coverage of the richest quintile, the NMR would have been reduced from 9.4 to 5.6 deaths per 1000 live births between 2012 and 2017 ^12^. International multicenter studies that included Peru showed that in the period 2000 to 2016 inequality in neonatal mortality related to wealth and maternal education decreased, but to still unacceptable levels ^13^ ^,^ ^14^.

This study aimed to analyze the inequality in the NMR between departments in Peru, generated by poverty and education, in the years 2011 and 2019.

**KEY MESSAGES**

Motivation for the study: The measurement of inequalities shows the existence, magnitude and trend of inequality in the population. Monitoring inequality enables the evaluation of intervention policies and programs from a social equity perspective.

Main findings: In Peru, more newborns die in poorer departments or in departments with less educated women. This inequality decreased between 2011 and 2019 reaching low magnitude values.

Implications: Maternal and neonatal health interventions should continue to be promoted, with a greater population focus, rather than focusing only on the poorest or least educated, leaving no one behind.

## MATERIALS AND METHODS

### Study design

An ecological study was conducted based on the departmental distribution of NMR in Peru according to poverty and education, in the years 2011 and 2019. We applied the method for analyzing health inequalities recommended by the World Health Organization ^15^ ^-^ ^17^.

### Health indicator

The health indicator we analyzed was the national and departmental NMR estimated by the Demographic and Family Health Survey (ENDES) for the years 2011 and 2019 ^18^ ^,^ ^19^. The ENDES is a population-based survey that explores content on reproductive health and child health among other important health topics and is conducted annually.

### Equity stratifiers

The selected equity stratifiers or social determinants of health were poverty and education. Structural poverty was considered before monetary poverty, because the purchasing power of the family alone does not define the risk of death in children; other conditions involved are housing characteristics, food, education and health car. Structural poverty was measured through the unsatisfied basic needs (UBN) method and the report of at least one UBN in the departmental population was considered. For this we used the map of unsatisfied basic needs of Peru 1993, 2007 and 2017, a document prepared with the results of the National Population Census 1993, 2007 and 2017 that were executed throughout the national territory and its jurisdictional waters after a census process ^20^. Education was defined as the average number of years of study of women of childbearing age, by department and reported by the ENDES, in years 2011 and 2019.

### Measurement of social inequalities in the NMR

Calculations were carried out with the Pan American Health Organization’s Equity Explorer ^21^. The departments were classified according to the equity stratifier, from greater to lesser poverty or from less to more education, for the years 2011 and 2019; they were grouped by quintiles, quintile 1 (Q1) represented the least favored quintile, with greater poverty or less education, and quintile 5 (Q5) was the most favored quintile. The weighted average of the NMR was calculated for each quintile, using the population of live births in each department as a weight.

We calculated simple and complex inequality metrics. As simple metrics, the absolute (AG) and relative (RG) inequality gaps were calculated by adding and dividing the NMR calculated for the extreme quintiles, Q1 and Q5.

As an inequality gradient metric, we calculated the health concentration index (HCI) to measure the degree of concentration of neonatal mortality among the most disadvantaged or the most advantaged according to the social stratifier, with values from -1 to +1; thus, the closer to zero the lower the inequality, a negative sign in the indicator indicated that the NMR was concentrated among the most disadvantaged. The HCI was represented by the concentration curve, which allowed us to analyze how far from a completely equal distribution the NMR was ^22^. Another complex inequality metric was the slope inequality index, which was discarded from the study because its calculation required applying a regression model that did not meet the basic requirements of normality and homoscedasticity in the health indicator; in addition, the calculated coefficients were not statistically significant, even when logarithmic transformations were applied.

This study was approved by the Ethics Committee of the Norbert Wiener University, file no. 089-2020.

## RESULTS

**Figure 1:**
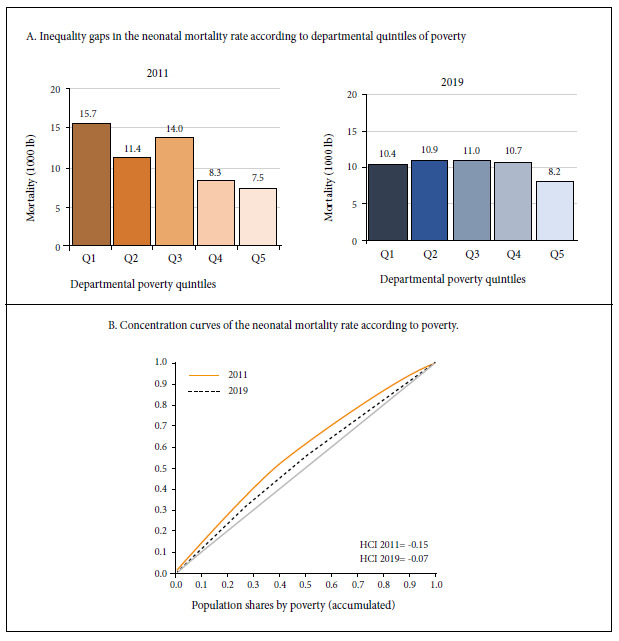
Inequalities in neonatal mortality generated by poverty.

Figure 1A shows the magnitude of inequality in neonatal mortality generated by poverty. In 2011, the average NMR in the Peruvian departments located in the highest poverty quintile Q1 (Huancavelica, Pasco, Loreto, Ucayali and San Martin) was 15.67 deaths per 1000 live births and in the departments located in the lowest poverty quintile Q5 (Moquegua, Lambayeque, Arequipa, Tacna and Lima) the average NMR was 7.54. The AG between the extreme quintiles Q1 and Q5 of 8.13 indicates that in 2011 the departments in the poorest quintile in Peru had eight more neonatal deaths per 1000 live births compared to the least poor quintile. The RG was 2.08, i.e., in 2011 neonatal mortality in the departments in the poorest quintile was twice the neonatal mortality for the departments of the least poor quintile. In 2019, the departments in the poorest quintile Q1 (Loreto, Ucayali, Amazonas, Pasco and San Martin) had an average NMR of 10.42 deaths per 1000 live births and in the departments located in the least poor quintile (Lambayeque, La Libertad, Arequipa, Tacna and Lima) the average NMR was 8.18 deaths per 1000 live births. The AG was 2.24 and the RG was 1.31. For the period 2011 and 2019, inequality was reduced by 72.4% for AG and 36.9% for RG.

The HCI was -0.15 in 2011 and -0.07 in 2019, reducing by 53%; negative inequality indicated higher concentration of neonatal deaths in the poorest departments. The concentration curve located above the diagonal showed that the poorest quintile departments accounted for 28% of neonatal deaths in 2011 and by 2019 inequality was reduced so neonatal deaths decreased to 21% in the poorest quintile (Figure 1B).

**Figure 2:**
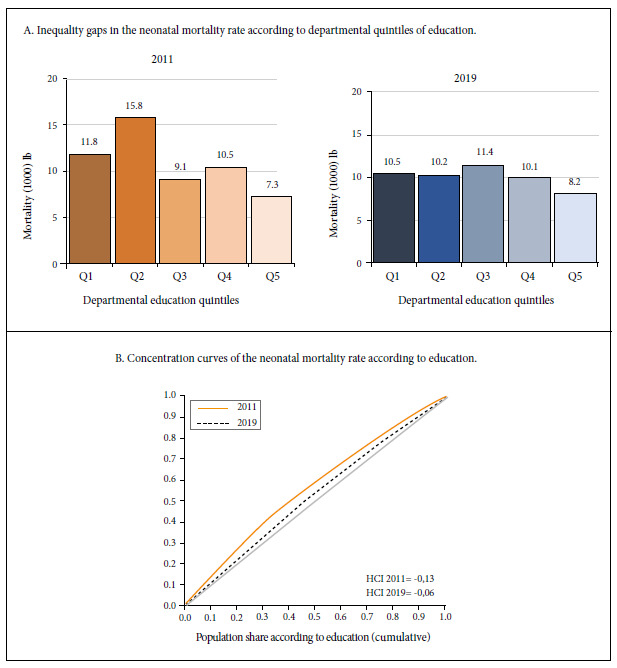
Inequalities in neonatal mortality generated by education.

Regarding inequality in neonatal mortality generated by education, the 2011 NMR for the departments in the quintile with the lowest education Q1 in women of childbearing age (Cajamarca, Huancavelica, Amazonas, Huánuco and Ayacucho) was 11.8 deaths per 1000 live births, while in the departments in the quintile with the best education in women of childbearing age (Tacna, Ica, Lima, Arequipa and Moquegua) the average NMR was 7.31. For 2019, the departments that ranked in Q1 were Cajamarca, Amazonas, Huánuco, San Martín and Loreto with an average NMR of 10.5 deaths per 1000 live births, while the departments in the quintile with the best education in women of childbearing age (Ica, Tacna, Lima, Moquegua and Arequipa) had an NMR of 8.2 deaths per 1000 live births. The AG decreased by 48.9% from 4.50 in 2011 to 2.31 excess deaths per 1000 live births in 2019, in addition, the RG was reduced by 20.9% from 1.62 to 1.28 (Figure 2A). The HCI in 2019 was negative and focused in the disadvantaged (less educated), decreasing by 53.8% since 2011, from -0.13 to -0.06. According to the concentration curves, in 2011 the departments located in the quintile with less education in women of childbearing age contributed about 25% of neonatal mortality, in 2019 the gradient decreased, so that the quintile with less education contributed about 21% of neonatal mortality (Figure 2B) (The data used for the analysis are included in the supplementary material).

## DISCUSSION

Our findings showed the existence of inequality in neonatal mortality generated by poverty and education in Peru in the years 2011 and 2019; however, this inequality was of lesser magnitude in 2019 and with a decreasing trend.

The analysis of inequality gaps in NMR generated by poverty showed that neonatal mortality was higher in the departments in the poorest quintiles compared to those in the least poor quintiles, mainly affecting the departments of Huancavelica, Loreto, Ucayali, Amazonas, Pasco and San Martin. Other studies conducted in the country also found that poverty affects the distribution of neonatal mortality ^12^ ^,^ ^23^ ^,^ ^24^. The NMR in the poorest quintile decreased by 34% between 2011 and 2019, but increased by 8% in the least poor quintile, which suggests an intensive targeting of interventions aimed at the poorest quintile, which should be extended to all quintiles. In addition, the pattern of behavior of inequality generated by poverty in 2011 was one of marginal exclusion, since the poorest quintile Q1 showed a higher NMR compared to the other four quintiles; by 2019 this pattern changed to mass deprivation, since the NMR was high in all quintiles except the poorest Q5, possibly as a result of excessive targeting of interventions aimed only at the poorest, leaving other population groups behind. This decreasing trend in absolute inequality coincides with studies carried out in Bolivia ^25^, Brazil ^26^, the United States ^27^ ^) ^and a multicenter study that included Peru ^28^.

If there were no social inequality and in 2019 the NMR between extreme quintiles according to poverty levels had been equal, 1268 neonatal deaths would have been avoided in Peru out of the 5570 that occurred according to estimates from the 2019 ENDES, that is, 22.7% of the neonatal mortality that occurred in 2019 would have been avoided; which is lower than what was found by Tam ^et al^, ^12^ who stated that if in 2017 the NMR between poor and non-poor groups had been equal, neonatal mortality could have been reduced by 41%.

Regarding the inequality generated by education, there is greater mortality in the departments of Cajamarca, Huancavelica, Amazonas, Huánuco, San Martín and Loreto, with a decreasing trend over time. Between 2011 and 2019 the NMR decreased by 11% in the least educated quintile and increased by 12% in the most educated quintile; in addition, the pattern of inequality behavior went from marginal exclusion in 2011 to mass deprivation in 2019, again evidencing that the priority were the interventions aimed at disadvantaged populations. Other studies have also shown that education is a social determinant of inequality that affects NMR ^28^ ^-^ ^30^. If in 2019 the average NMR of the departments in the extreme quintiles, according to maternal education, had been equal (absolute equity assumption), 1268 neonatal deaths would have been avoided in Peru.

Between 2011 and 2019, the HCI decreased close to zero and the concentration curves approached the equity line (diagonalization), coinciding with the results of studies conducted in Bolivia ^25^ and in low- and middle-income countries ^13^.

This is the most updated national study on the analysis of social inequalities in neonatal mortality, which incorporated poverty and education among the equity stratifiers. The methodology for analyzing health inequalities is a useful tool for highlighting and monitoring inequities and improving decision-making in health equity, but it is not included in the situation analysis tools developed by the Ministry of Health for the analysis of the country’s health situation. Furthermore, Peru does not have an observatory on social inequalities like Colombia or Mexico, for example.

The limitations of the study are linked to the ecological design, which does not allow causality to be inferred; however, our results can serve as the basis for future research by using more robust methodological designs for the analysis of neonatal mortality and the existing disparities in its distribution. Another limitation was having a static national NMR since 2014 and one without changes at the departmental level since 2017. Due to the fact that the estimates made by the ENDES are shown in ranges (due to high coefficients of variation) and do not vary, they do not allow us to objectively assess the trend of inequality; these surveys are the official statistics reported by the country and used for international comparison. Neonatal mortality was analyzed at the departmental level as the maximum level of territorial disaggregation, since it is difficult to access information on NMR at lower levels such as provinces or districts, which requires organizing quintiles with provinces or districts that could belong to a different poverty or education quintile, a situation that could alter the results of the measures of inequality gaps and gradients.

In conclusion, we found inequality in neonatal mortality between departments in Peru, in years 2011 and 2019, generated by poverty and education. More newborns die in departments with greater poverty or with a lower level of education. This inequality is of low magnitude, reaching values close to zero in the social gradient metrics. It is recommended that maternal and neonatal health interventions be developed with a greater focus on the whole population, rather than focusing on the poorest or least educated, given that the NMR in the least favored quintiles decreased between 2011 and 2019, but increased in the most favored quintiles. The Peruvian state should continue to demonstrate the existence of inequality and facilitate access to this information to allow the implementation of better informed and more effective policies, which will serve as a basis for investing in the health of children from disadvantaged or socially excluded families in order to reduce inequality to low and socially tolerable levels. It is also recommended that a national observatory for the analysis of inequalities in maternal and neonatal health be implemented in order to provide more and better evidence to reduce inequality and promote equity, in compliance with the Sustainable Development Goals. There is an urgent need to improve access to disaggregated data and monitoring of newborn health in order to identify the populations that are in a situation of greater social exclusion. Improving health implies the inclusion of all, leaving no one behind.

## References

[1] Mendoza Nava A (2019). Brechas latentes. Índice de avance contra la desigualdad en el Perú 2017-2018.

[2] United Nations International Children's Emergency Fund (2016). Estado Mundial de la Infancia 2016: Una oportunidad para cada niño.

[3] United Nations International Children's Emergency Fund (2017). Reducir las diferencias:el poder de invertir en los niños más pobres.

[4] Perova E, Vakis R (2012). 5 Years in Juntos New Evidence on the Program's Short and Long-Term Impacts. Economia [Internet].

[5] Cotlear D, Vermeersch C (2016). Peruvian lessons for the transition from MDGs to SDGs. Lancet Glob Health.

[6] Huicho L, Segura ER, Huayanay-Espinoza CA, Niño De Guzmán J, Restrepo-Méndez MC, Tam Y (2016). Child health and nutrition in Peru within an antipoverty political agenda a Countdown to 2015 country case study. Lancet Glob Health [Internet].

[7] Ávila J (2020). Mortalidad neonatal problema prioritario de salud pública por resolver. An Fac med.

[8] United Nations (2015). Transforming our world: the 2030 Agenda for Sustainable Development Transforming our world: the 2030 Agenda for Sustainable Development Preamble.

[9] UNICEF (2019). Identificar las desigualdades para actuar: Resultados y determinantes del Desarrollo de la Primera Infancia en América Latina y el Caribe.

[10] Victora CG, Barros AJD (2014). Socioeconomic inequalities in neonatal mortality are falling but why?. Lancet Glob Health.

[11] World Health Organization (2020). Newborns: improving survival and well-being.

[12] Tam Y, Huicho L, Huayanay-Espinoza CA, Restrepo-Méndez MC (2016). Remaining missed opportunities of child survival in Peru: Modelling mortality impact of universal and equitable coverage of proven interventions. BMC Public Health.

[13] McKinnon B, Harper S, Kaufman JS, Bergevin Y (2014). Socioeconomic inequality in neonatal mortality in countries of low and middle income A multicountry analysis. Lancet Glob Health.

[14] Sanhueza A, Carvajal-Vélez L, Mújica OJ, Vidaletti LP, Victora CG, Barros AJD (2021). SDG3-related inequalities in women's, children's and adolescents' health An SDG monitoring baseline for Latin America and the Caribbean using national cross-sectional surveys. BMJ Open.

[15] World Health Organization (2017). National Health Inequality Monitoring. A step by step manual.

[16] World Health Organization (2015). Monitoring Health Inequality. An essential step for achieving health equity.

[17] Schneider MC, Castillo-Salgado C, Bacallao J, Loyola E, Mujica OJ, Vidaurre M (2002). Métodos de medición de las desigualdades de salud. Rev Panam Salud Publica.

[18] Instituto Nacional de Estadística e Informática (2011). Encuesta Demográfica de Salud Familiar 2011.

[19] Instituto Nacional de Estadística e Informática (2020). Perú: Encuesta Demográfica y de Salud Familiar 2019.

[20] Instituto Nacional de Estadística e Informática (2018). PERÚ: Mapa de Necesidades Básicas Insatisfechas (NBI), 1993, 2007 y 2017.

[21] Mujica OJ (2016). Explorador de Equidad: plantilla OPS para el análisis exploratorio de datos sobre desigualdades sociales en salud.

[22] Mújica ÓJ, Moreno CM (2019). De la retórica a la acción: medir desigualdades en salud para "no dejar a nadie atrás". Rev Panam Salud Publica.

[23] Huicho L, Huayanay-Espinoza CA, Herrera-Pérez E, Niño De Guzmán J, Rivera-Ch M, Restrepo-Méndez MC (2016). Examining national and district-level trends in neonatal health in Peru through an equity lens: A success story driven by political will and societal advocacy. BMC Public Health.

[24] Paredes R, Yapuchura C, Arpi R, Calatayud A (2021). Determinantes socioeconómicos y próximos de la mortalidad de niños menores de cinco años en el Perú (2015-2018). Apuntes.

[25] Alarcón WR (2017). Factores socioeconómicos y zona de residencia como estratificadores de desigualdades en salud en Bolivia. Rev Panam Salud Publica.

[26] Menezes AMB, Barros FC, Horta BL, Matijasevich A, Bertoldi AD, Oliveira PD (2019). Stillbirth, newborn and infant mortality Trends and inequalities in four population-based birth cohorts in Pelotas, Brazil, 1982-2015. Int J Epidemiol.

[27] Turner N, Danesh K, Moran K (2020). The evolution of infant mortality inequality in the United States, 1960-2016. Science Advances.

[28] Lohela TJ, Nesbitt RC, Pekkanen J, Gabrysch S (2019). Comparing socioeconomic inequalities between early neonatal mortality and facility delivery Cross-sectional data from 72 low- and middle-income countries. Scientific Reports.

[29] Tullo E, Lerea MJ, González R, Galeano J, Insfrán MD, Muñoz M (2020). Desigualdades sanitarias y sociales en la salud materna y del niño en Paraguay. Rev Panam Salud Publica.

[30] Aguilera X, Delgado I, Icaza G, Apablaza M, Villanueva L, Castillo-Laborde C (2020). Under five and infant mortality in Chile (1990-2016): Trends, disparities, and causes of death. PLoS ONE.

