# Supplementary material for: Inequality in Peruvian neonatal mortality generated by poverty and education, 2011-2019
Source: Rev Peru Med Exp Salud Publica. 2022 Jun 30;39(2):178–84. doi: 10.17843/rpmesp.2022.392.10629 (PMC11397582; doi:10.17843/rpmesp.2022.392.10629)
Supplement: Translation

# Translation: Desigualdad en la mortalidad neonatal del Perú generada por la pobreza y educación, 2011-2019

**Authors:** Jeannette Giselle Ávila Vargas-Machuca

**Affiliations:** 1 Centro Nacional de Epidemiologia, Prevención y Control de Enfermedades del Ministerio de Salud, Lima, Perú.

**Keywords:** Inequidad Social, Determinantes Sociales de la Salud, Escolaridad, Pobreza, Perú

## Abstract

**Objetivo:**

El objetivo de este estudio fue analizar la desigualdad en la TMN entre departamentos del Perú, generada por la pobreza y educación, en los años 2011 y 2019.

**Materiales y métodos:**

Estudio ecológico basado en el análisis de desigualdades sociales en salud recomendada por la Organización Mundial de la Salud. El indicador de salud fue la TMN y como estratificadores de equidad se seleccionaron a la pobreza, medida como la existencia de al menos una necesidad básica insatisfecha por departamento, y a la educación, promedio de años de estudio de las mujeres en edad fértil por departamento. Se calculó la desigualdad absoluta (BA), relativa (BR) y el índice de concentración de salud (ICS).

**Resultados:**

Se encontró mayor TMN en departamentos con mayor pobreza y menor educación. En la TMN generada por la pobreza la BA se redujo de 8,13 a 2,24 entre 2011-2019 y la BR de 2,08 a 1,31. La BA de la TMN según educación pasó de 4,50 a 2,31 y la BR de 1,62 a 1,28. El ICS registró valores cercanos a cero y con tendencia a la reducción; en el 2019 fue 0,07 para la pobreza y 0,06 para la educación.

**Conclusiones:**

Existe desigualdad en la mortalidad neonatal entre departamentos del Perú según pobreza y la educación, habiéndose reducido entre el 2011 y 2019 principalmente en la población pobre o menos educada. El Ministerio de Salud debe continuar reduciendo la mortalidad neonatal impulsando intervenciones con mayor enfoque poblacional.

## INTRODUCCIÓN

El Perú es un país de grandes desigualdades donde existen grupos bien diferenciados, ya sea por su ubicación geográfica o por su accesibilidad a recursos económicos y políticos. Las poblaciones relegadas se caracterizan por ser las más pobres, procedentes de zonas rurales, menos educadas, indígenas y con otras condiciones sociales desfavorables con menores posibilidades de tener una atención de salud cuando lo necesitan, acceder a educación de calidad, al trabajo o a la vivienda digna ^1^. Las desigualdades sociales generan brechas en las posibilidades de sobrevivencia de los recién nacidos, frenando el avance sostenible que busca que los niños puedan ejercer su derecho a sobrevivir y crecer ^2^ ^,^ ^3^.

El Perú tuvo importantes avances en la reducción de la mortalidad infantil hasta el año 2013 lo que le permitió el cumplimiento de los Objetivos de Desarrollo del Milenio, ello gracias a la mejora de la cobertura de intervenciones específicas como planificación familiar, atención prenatal y asistencia calificada del parto, las que se incrementaron en zonas de pobreza y rurales; además del crecimiento económico del Perú y reducción de la pobreza entre 1990 y 2010 ^4^ ^-^ ^6^.

Actualmente el descenso de la mortalidad infantil es lento y el componente de la mortalidad neonatal es el más predominante. La muerte neonatal es un evento que ocurre con alta frecuencia, más de 5500 defunciones anuales a nivel nacional, donde el 30% de las defunciones ocurren en recién nacidos de buen peso, a término y sin malformaciones congénitas letales (mortalidad neonatal evitable). Además, la tasa de mortalidad neonatal (TMN) nacional está estancada en 10 por 1000 nacidos vivos desde el año 2014 y es mayor en departamentos con mayor pobreza, menor educación, mayor ruralidad y departamentos ubicados en regiones de la selva y sierra central ^7^ por lo que este evento requiere ser abordado con enfoque de equidad en las políticas públicas y programas nacionales.

El objetivo 3 de los Objetivos de Desarrollo Sostenible «Garantizar una vida sana y promover el bienestar de todos a todas las edades» está dirigido a asegurar la salud y el bienestar mediante la mejora de la salud reproductiva, materna e infantil, entre otros daños, sin dejar a nadie atrás y buscando construir una verdadera alianza para el desarrollo donde todos los países participen, por lo que sugiere medir las desigualdades en los indicadores de salud, monitorear su tendencia en el tiempo y establecer estrategias para promover la equidad ^8^ ^,^ ^9^. El monitoreo continuo de las desigualdades en la TMN y la cobertura de intervención del recién nacido debe ser un aspecto clave de cualquier estrategia para llegar a todas las madres y sus recién nacidos ^10^ ^,^ ^11^.

Estudios nacionales previos exploraron la desigualdad en la mortalidad neonatal generada por la pobreza, mas no evaluaron la educación. Así, tenemos a Huicho *et al*, quienes encontraron que en el período 1999-2001, la TMN para los quintiles menos pobres y más pobres fue de 7,8 y 21,9 muertes por 1000 nacidos vivos, respectivamente, mientras que para el período 2011-2013 la TMN fue 9,1 y 11, evidenciando que en grupos más pobres y rurales la TMN tuvo mayor reducción dado el crecimiento económico y la disminución de la pobreza en el Perú ^6^. Tam *et al*, estimaron la TMN al 2017 eliminando las desigualdades en la distribución de cobertura de las intervenciones de salud materna, neonatal e infantil entre los quintiles de riqueza, encontrando que, si la cobertura del quintil más pobre fuera igual a la cobertura del quintil más rico, la TMN se habría reducido de 9,4 a 5,6 muertes por 1000 nacidos vivos entre el 2012 y 2017 ^12^. Estudios internacionales multicéntricos que incluyeron a Perú entre los países participantes demostraron que en el periodo 2000 al 2016 la desigualdad en la mortalidad neonatal relacionada con la riqueza y educación materna disminuyó, pero a niveles aún inaceptables ^13^ ^,^ ^14^.

El objetivo de este estudio fue analizar la desigualdad en la TMN entre departamentos del Perú, generada por la pobreza y educación, en los años 2011 y 2019.

**MENSAJE CLAVE**

Motivación para realizar el estudio: la medición de desigualdades evidencia la existencia, magnitud y tendencia de la desigualdad en la población. El monitoreo de la desigualdad permite evaluar las políticas y programas de intervención con una mirada de equidad social.

Principales hallazgos: en el Perú fallecen más neonatos en departamentos más pobres o con menor educación en sus mujeres. Esta desigualdad se redujo entre el 2011 y 2019 alcanzando valores de baja magnitud.

Implicancias: se debe continuar impulsando intervenciones de salud materno neonatal, con mayor enfoque poblacional, más que focalizado solo a los más pobres o los menos educados, sin dejar a nadie atrás.

## MATERIALES Y MÉTODOS

### Diseño del estudio

Se realizó un estudio ecológico basado en la distribución departamental de la TMN en el Perú según pobreza y educación, en los años 2011 y 2019. Se aplicó la metodología de análisis de desigualdades en salud recomendada por la Organización Mundial de la Salud ^15^ ^-^ ^17^.

### Indicador de salud

El indicador de salud analizado fue la TMN nacional y departamental estimada por la encuesta demográfica y de salud familiar (ENDES) de los años 2011 y 2019 ^18^ ^,^ ^19^. La ENDES es una encuesta poblacional que explora contenidos sobre salud reproductiva y salud infantil entre otros temas importantes de salud y se realiza anualmente. 

### Estratificadores de equidad

Los estratificadores de equidad o determinantes sociales de la salud seleccionados fueron pobreza y educación. Se consideró la pobreza estructural antes que la pobreza monetaria, dado que la capacidad adquisitiva del hogar por sí sola no define el riesgo de muerte en los niños, influyen otras condiciones de vida como las características de la vivienda, la alimentación, la educación y la atención de salud, por lo que la pobreza estructural se midió a través del método de necesidades básicas insatisfechas (NBI) y se consideró el reporte de la existencia de al menos un NBI en la población departamental. La fuente utilizada fue el mapa de necesidades básicas insatisfechas del Perú 1993, 2007 y 2017, documento elaborado con los resultados del Censo Nacional de Población 1993, 2007 y 2017 que se ejecutaron en todo el territorio nacional y sus aguas jurisdiccionales luego de un proceso de empadronamiento ^20^. La educación se definió como el promedio de años de estudio de las mujeres en edad fértil, por departamento y reportada por la ENDES, años 2011 y 2019.

### Medición de desigualdades sociales en la TMN

Los cálculos se realizaron con el Explorador de Equidad de Organización Panamericana de Salud ^21^. Los departamentos se ordenaron en función al estratificador de equidad, de mayor a menor pobreza o de menor a mayor educación, para los años 2011 y 2019, y se agruparon por quintiles; así, el quintil 1 (Q1) representó al quintil menos favorecido, con mayor pobreza o menor educación y quintil 5 (Q5) fue el quintil más favorecido. Se calculó el promedio ponderado de la TMN para cada quintil, utilizando como ponderador la población de nacidos vivos de cada departamento.

Se calcularon métricas de desigualdad simples y complejas. Como métricas simples se calcularon las brechas de desigualdad absoluta (BA) y relativa (BR) que se obtuvieron sumando y dividiendo las TMN calculadas para los quintiles extremos, Q1 y Q5.

Como métrica de gradiente de desigualdad se calculó el índice de concentración en salud (ICS) que midió el grado de concentración de la mortalidad neonatal entre los más desaventajados o los más aventajados de acuerdo con el estratificador social estudiado, con valores de -1 a +1; así, cuanto más cercano a cero menor desigualdad, un signo negativo en el indicador indicó que la TMN se concentró entre los más desaventajados. El ICS se representó a través de la curva de concentración que permitió analizar cuán lejos de una distribución completamente equitativa se encontró la TMN ^22^. Otra métrica compleja de desigualdad evaluada fue el índice de desigualdad de la pendiente, que se descartó del estudio porque su cálculo requería aplicar un modelo de regresión no cumplía con los requisitos básicos de normalidad y homocedasticidad en el indicador de salud; además, los coeficientes calculados no fueron estadísticamente significativos, aun cuando se hicieron transformaciones logarítmicas.

Este estudio fue aprobado por el Comité de Ética de la Universidad Norbert Wiener, expediente n.° 089-2020.

## RESULTADOS

En la figura 1A se observa la magnitud de la desigualdad en la mortalidad neonatal generada por la pobreza. En el año 2011 la TMN promedio en los departamentos del Perú ubicados en el quintil con mayor pobreza Q1 (Huancavelica, Pasco, Loreto, Ucayali y San Martín) fue de 15,67 muertes por 1000 nacidos vivos y en los departamentos ubicados en el quintil con menor pobreza Q5 (Moquegua, Lambayeque, Arequipa, Tacna y Lima) la TMN promedio fue 7,54. La BA entre los quintiles extremos Q1 y Q5 de 8,13 indica que en el 2011 los departamentos ubicados en el quintil más pobre del Perú tuvieron ocho muertes neonatales más por cada 1000 nacidos vivos en comparación al quintil menos pobre. La BR fue 2,08, es decir, en el 2011 la mortalidad neonatal en los departamentos del quintil más pobre fue dos veces la mortalidad neonatal registrada en los departamentos del quintil menos pobre. En el 2019, los departamentos del quintil más pobre Q1 (Loreto, Ucayali, Amazonas, Pasco y San Martín) tuvieron una TMN promedio fue 10,42 muertes por 1000 nacidos vivos y en los departamentos ubicados en el quintil menos pobre (Lambayeque, La Libertad, Arequipa, Tacna y Lima) la TMN promedio fue 8,18 muertes por 1000 nacidos vivos. La BA fue 2,24 y la BR fue 1,31. Para el periodo 2011 y 2019 la desigualdad se redujo en 72,4% para la BA y en 36,9% para la BR.

**Figura 1:** Desigualdades de la mortalidad neonatal generada por la pobreza.

El ICS fue -0,15 en 2011 y pasó a -0,07 en 2019 reduciéndose en 53%, la desigualdad negativa indicó mayor concentración de muertes neonatales en los departamentos más pobres. La curva de concentración ubicada por encima de la diagonal indicó que los departamentos del quintil más pobre concentraron el 28% de las muertes neonatales en el 2011 y para 2019 la desigualdad se redujo de forma que el quintil más pobre acumuló cerca del 21% de las muertes neonatales (Figura 1B).

Con respecto a la desigualdad en la mortalidad neonatal generada por la educación, la TMN 2011 en los departamentos ubicados en el quintil con menor educación Q1 en mujeres en edad fértil (Cajamarca, Huancavelica, Amazonas, Huánuco y Ayacucho) fue de 11,8 muertes por 1000 nacidos vivos, mientras que en los departamentos ubicados en el quintil con mejor educación en mujeres en edad fértil (Tacna, Ica, Lima, Arequipa y Moquegua) la TMN promedio fue 7,31. Para el año 2019 los departamentos que se ubicaron en el Q1 fueron Cajamarca, Amazonas, Huánuco, San Martín y Loreto con una TMN promedio de 10,5 muertes por 1000 nacidos vivos, mientras que en los departamentos ubicados en el quintil con mejor educación en mujeres en edad fértil (Ica, Tacna, Lima, Moquegua y Arequipa) la TMN fue 8,2 muertes por 1000 nacidos vivos. La BA se redujo en 48,9% pasando de 4,50 el 2011 a 2,31 muertes en exceso por cada 1000 nacidos vivos en el 2019, además, la BR se redujo en 20,9% pasando de 1,62 a 1,28 (Figura 2A). El ICS en el 2019 fue negativo y se concentró en los desaventajados (menos nivel educativo), reduciéndose en 53,8% desde el 2011, pasando de -0,13 a -0,06. Según las curvas de concentración, en el 2011 los departamentos ubicados en el quintil con menor educación en mujeres en edad fértil aportaron cerca del 25% de la mortalidad neonatal, en el 2019 la gradiente disminuyó, por lo que el quintil con menor educación aportó cerca del 21% de la mortalidad neonatal (Figura 2B) (Los datos utilizados para el análisis se adjuntan en el material suplementario).

**Figura 2:** Desigualdades de la mortalidad neonatal generada por la educación.

## DISCUSIÓN

Los hallazgos de este estudio evidenciaron la existencia de desigualdad en la mortalidad neonatal generada por la pobreza y educación en el Perú en los años 2011 y 2019, sin embargo, esta desigualdad fue de menor magnitud en el 2019 y con tendencia a la reducción.

El análisis de brechas de desigualdad de la TMN generada por la pobreza evidenció mayor mortalidad neonatal en los departamentos ubicados en quintiles más pobres en comparación a los menos pobres, afectando principalmente a los departamentos de Huancavelica, Loreto, Ucayali, Amazonas, Pasco y San Martín. Otros estudios realizados en el país también encontraron que la pobreza afecta la distribución de la mortalidad neonatal ^12^ ^,^ ^23^ ^,^ ^24^. La TMN en el quintil más pobre se redujo en 34% entre 2011 y 2019, pero en el quintil menos pobre se incrementó en 8% lo cual hace referencia a una focalización intensiva de las intervenciones a predominio del quintil más pobre, debiéndose ampliar las intervenciones a todos los quintiles. Además, el patrón de comportamiento de la desigualdad generada por la pobreza para el año 2011 fue de exclusión marginal, pues el quintil más pobre Q1 mostró una mayor TMN comparada a los otros cuatro quintiles; para el 2019 este patrón cambia a deprivación en masa, dado que la TMN fue alta en todos los quintiles a excepción del Q5 menos pobre, posiblemente resultado de una excesiva focalización de intervenciones dirigidas solo a los más pobres dejando atrás a otros grupos poblacionales. Esta tendencia de reducción de la desigualdad absoluta coincide con estudios realizados en Bolivia ^25^, Brasil ^26^, Estados Unidos ^27^ y un estudio multicéntrico donde se incluyó a Perú ^28^.

Si no existiera desigualdad social y en el año 2019 las TMN entre quintiles extremos según niveles de pobreza hubiesen sido iguales, se habrían evitado 1268 muertes neonatales en el Perú de las 5570 que ocurrieron según estimaciones de la ENDES 2019, es decir, se habría evitado el 22,7% de la mortalidad neonatal ocurrida en el 2019; valor menor al encontrado por Tam *et al*, ^12^ que afirman que si en el 2017 la TMN entre grupos pobres y no pobres hubiese sido igual, la mortalidad neonatal podría haberse reducido en un 41%.

Con respecto a la desigualdad generada por la educación, se evidencia mayor mortalidad en los departamentos de Cajamarca, Huancavelica, Amazonas, Huánuco, San Martín y Loreto, con una tendencia a la reducción en el tiempo. Entre los años 2011 y 2019 hubo un descenso de 11% en la TMN del quintil menos educado y un aumento de 12% en la TMN del quintil más educado; además, el patrón de comportamiento de la desigualdad pasó de exclusión marginal en 2011 a deprivación en masa en 2019, evidenciándose nuevamente la focalización de intervenciones en poblaciones desfavorecidas prioritariamente. Otros estudios también demostraron que la educación es un determinante social de la desigualdad que afecta la TMN ^28^ ^-^ ^30^. Si en el 2019 la TMN promedio de los departamentos ubicados en los quintiles extremos según educación materna hubiesen sido iguales (supuesto de equidad absoluta), se habrían evitado 1268 muertes neonatales en el Perú.

Entre los años 2011 y 2019 se redujo el ICS con valores cercanos a cero y las curvas de concentración se aproximaron hacia la línea de equidad (diagonalización), coincidiendo con resultados de estudios realizados en Bolivia ^25^ y países de bajos y medianos ingresos ^13^.

Este es el estudio nacional más actualizado sobre análisis de las desigualdades sociales en la mortalidad neonatal, que incorpora dentro de los estratificadores de equidad a la pobreza y educación. La metodología de análisis de desigualdades de salud es una herramienta útil para evidenciar y monitorear las inequidades y avanzar en la toma de decisiones en equidad en salud, pero no está incluida en las herramientas de análisis de situación que desarrolla el Ministerio de Salud para el análisis de situación de salud del país. Además, el Perú no cuenta con un observatorio en desigualdades sociales como Colombia o México, por ejemplo.

Las limitaciones del estudio están vinculadas al diseño ecológico, que no permite inferir causalidad, sin embargo, sus resultados pueden servir como base para futuras investigaciones que utilicen diseños metodológicos más robustos para el análisis de la mortalidad neonatal y las disparidades existentes en su distribución. Otra limitación fue disponer de una TMN nacional estática desde el 2014 y en el nivel departamental sin cambios desde el año 2017, debido a que las estimaciones realizadas por la ENDES se muestran por rangos (debido a elevados coeficientes de variación), no varían y, por ende, no permiten valorar objetivamente la tendencia de la desigualdad, aun así, estas encuestas son las estadísticas oficiales que reporta el país y que se utilizan para la comparación internacional. El máximo nivel de disgregación territorial aplicado en el análisis de la mortalidad neonatal es el departamental, ya que en el país existen dificultades para acceder a información de TMN en niveles inferiores como provincias o distritos, lo cual obliga a organizar quintiles donde se ubican provincias o distritos que podrían pertenecer a un quintil diferente de pobreza o educación, situación que podría alterar los resultados de las medidas de brechas y gradientes de desigualdad.

En conclusión, existe desigualdad en la mortalidad neonatal entre departamentos del Perú, entre los años 2011 y 2019 generada por la pobreza y la educación. Fallecen más neonatos en departamentos donde existe mayor pobreza o menor nivel educativo. Esta desigualdad es de baja magnitud, llegando a valores cercanos a cero en las métricas de gradiente social. Se recomienda desarrollar las intervenciones de salud materno neonatal con mayor enfoque poblacional, más que focalizado en los más pobres o los menos educados, dado que las TMN en quintiles menos favorecidos se redujo entre 2011 y 2019, caso contrario se incrementó en los quintiles más favorecidos. El estado peruano debe continuar evidenciando la existencia de la desigualdad y facilitar el acceso a esta información para permitir la adopción de políticas mejor informadas y más efectivas, que sirvan de sustento para invertir en la salud de los niños procedentes de familias desfavorecidas o excluidas socialmente a fin de disminuir la desigualdad a niveles bajos y socialmente tolerables. Se recomienda la implementación de un observatorio nacional de análisis de desigualdades en salud materna y neonatal que permita generar mayor y mejor evidencia en la reducción de la desigualdad y favorecer la equidad, en cumplimiento de los Objetivos de Desarrollo Sostenible. Urge mejorar el acceso a datos desagregados y el monitoreo en salud del neonato a fin de identificar aquellas poblaciones que se encuentran en situación de mayor exclusión social. La mejora de salud implica la inclusión de todos, sin dejar a nadie atrás.
